# Use of Wild Ungulates as Sentinels of TBEV Circulation in a Naïve Area of the Northwestern Alps, Italy

**DOI:** 10.3390/life12111888

**Published:** 2022-11-15

**Authors:** Aitor Garcia-Vozmediano, Alessandro Bellato, Luca Rossi, Marieke N. Hoogerwerf, Hein Sprong, Laura Tomassone

**Affiliations:** 1 Department of Veterinary Sciences, University of Turin, L.go Braccini, 2, 10095 Grugliasco, TO, Italy; 2 Centre for Infectious Disease Control, National Institute for Public Health and the Environment, Antonie van Leeuwenhoeklaan 9, 3720 MA Bilthoven, The Netherlands

**Keywords:** tick-borne encephalitis virus, serology, *Cervus elaphus*, *Rupicapra rupicapra*, *Capreolus capreolus*, northwestern Italy

## Abstract

Wild and domestic animals can be usefully employed as sentinels for the surveillance of diseases with an impact on public health. In the case of tick-borne encephalitis virus (TBEV), the detection of antibodies in animals can be more effective than screening ticks for detecting TBEV foci, due to the patchy distribution of the virus. In the Piedmont region, northwestern Italy, TBEV is considered absent, but an increase in tick densities, of *Ixodes ricinus* in particular, has been observed, and TBEV is spreading in bordering countries, e.g., Switzerland. Therefore, we collected sera from wild ungulates during the hunting season (October–December) from 2017 to 2019 in the Susa Valley, Italian western Alps, and screened them for TBEV antibodies by a commercial competitive ELISA test. We collected 267 serum samples by endocranial venous sinuses puncture from red deer, roe deer and northern chamois carcasses. The animals were hunted in 13 different municipalities, at altitudes ranging between 750 and 2800 m a.s.l. The serological survey for TBEV yielded negative results. Borderline results for five serum samples were further confirmed as negative for TBEV by a plaque reduction neutralisation test. To date, our results indicate that TBEV is not circulating in western Piedmont. However, monitoring of TBEV should continue since TBEV and its vector are spreading in Europe. The wide-range distribution of wild ungulates and their role as feeding hosts, make them useful indicators of the health threats posed by Ixodid ticks.

## 1. Introduction

Increasing interactions at the human–animal–environment interface are associated with the occurrence of emerging infectious diseases [1]. Wildlife, in particular, historically played a major role as a source or reservoir of several emerging diseases threatening both animal and human health [2]. This fact has enabled the use of wildlife as sentinels of emerging health threats, including tick-borne diseases. Passive surveillance activities on wildlife, for instance, allowed detection of the Crimean-Congo haemorrhagic fever virus emergence in ticks engorged on red deer in Spain [3], and tick-borne encephalitis virus (TBEV) in ticks and roe deer in the Netherlands and the United Kingdom [4,5] prior to the occurrence of human cases [6,7,8]. Moreover, wildlife can provide data about the potential geographic distribution of tick vectors and transmitted pathogens.

Among the broad range of pathogens transmitted by *Ixodes* ticks, TBEV is a tick-borne flavivirus of major concern for public health in Europe, and its incidence is increasing [9]. *Ixodes ricinus*, together with small rodents and insectivores, plays a major role in the maintenance of TBE natural foci in western Europe [10,11]. TBEV infections in humans can result in severe complications, associated with neurological disorders of variable degrees, sometimes causing long-lasting sequelae or even death [12,13]. This flavivirus is mainly contracted through infected tick bites, although alternative infection routes have been described, such as the ingestion of infected raw milk and dairy products or organ transplants [14].

Since its first discovery in the late 1930s, five different TBEV subtypes have been identified, differing in their geographic distribution in Europe and Asia [15,16,17]. The European (TBEV-Eu), Siberian (TBEV-Sib) and Far Eastern (TBEV-FE) are the main subtypes circulating in Europe, with the predominance of TBEV-Eu. In some geographic contexts, however, the three subtypes coexist, which is consistent with the overlapped distribution of their main competent tick vectors—i.e., *Ixodes ricinus* for TBEV-Eu and *I. persulcatus* for TBEV-Sib and TBEV-FE [18]. These tick species have increased their geographic distribution in Europe, especially *I. ricinus*, leading to an increase in associated tick-borne diseases. Accordingly, several endemic European countries have experienced a general upward trend in human TBE cases [19,20]. The combination of socioeconomic factors, global warming, and changes in the landscape seems to be the most plausible driver for the distribution of TBEV [11,21,22], by enhancing suitable areas for its establishment and spread. Novel natural foci of TBEV have been steadily reported across Europe following different trends of spread. Phylogeographic studies have uncovered east-to-west shifts in central Europe, with the identification of closely related viral strains circulating even across long distances and suggesting a common geographic origin [23,24]. For instance, TBEV has recently jumped out from continental Europe to colonize southern forest areas of the United Kingdom, where the identified viral strains are similar to strains recently circulating in central Europe [5]. At its northern limits, TBEV has spread northwards, causing disease cases in the resident populations in areas thought to be free of TBEV circulation [25,26,27], and new natural foci are still emerging [28,29]. In mountain areas, TBEV occurrence has also experienced gradual changes in its altitudinal limit, moving upwards to altitudes up to 1564 m a.s.l. [30,31,32,33]. In the Alpine arch, shifts in TBEV endemicity have been also observed in the last 20 years in Switzerland [34,35,36,37] and France [38,39]. Indeed, a significant increase (48–88%) in TBE cases was highlighted in Alpine regions in 2020, compared with 2017–2019 [9]. For instance, Switzerland’s Federal Office of Public Health has recorded a general increase of the disease in the resident population, registering incidence rates of 1.16/100,000 inhabitants in 2012 and 3.25/100,000 in 2021 [40]. Seroprevalence studies performed in goat flocks have also disclosed new natural foci of TBEV in the Swiss Alps, with prevalence ranging between 4.3% and 14.6% [36,37]. In France, the incidence of TBE in humans is very low (0.5/100,000 inhabitants), with cases mainly distributed in eastern areas [39,41]. Recent TBE alimentary outbreaks have been detected in naïve eastern French areas, where epidemiological investigations ascertained a seroprevalence between 5.5 and 25.0% in livestock [39].

In Italy, TBEV is historically present in northeastern regions [42,43]. The incidence is relatively low; in 2017–2019, on average 33 cases were confirmed, corresponding to around a 0.1% infection rate per 100,000 inhabitants [44]. However, TBE incidence in humans has been gradually increasing over the years [45], and recently, a TBE case was reported in a non-endemic area in the Emilian Apennines, central Italy [46]. By contrast, no local human cases of TBE have been detected to date in the northwestern regions where ticks are a more recent threat [47]. This leads to the assumption that TBEV is absent in the territory. However, the recent expansion of *I. ricinus* in the northwestern Alps alongside the circulation of TBEV in neighbouring countries, such as France and Switzerland [36,37,38], highlights the risk of its introduction. 

Therefore, we investigated the possible occurrence of TBEV in an Alpine valley of the Piedmont region, northwestern Italy, by testing hunted wild ungulates for the presence of TBEV antibodies. Wild ungulates are important hosts for ticks and the fact that they develop a long-lasting immune response can enable the tracing of TBEV exposure over time [48], helping in the identification of potential risk areas.

## 2. Materials and Methods

### 2.1. Blood Samples Collection from Wild Ungulates

Serum samples were collected from red deer, roe deer and northern chamois during three consecutive hunting seasons, from 2017 to 2019 (September to December). As per regulations in force, hunters are bound to present this game on the culling day at the check station of the local Hunting Unit (CA TO2) in Susa Valley, Piedmont. The Piedmont region is an Alpine region bordering France in the east and Switzerland in the north.

Blood samples (5 mL maximum when possible) were taken through endocranial venous sinuses puncture, as previously described [49], using a 105-mm-long needle (2.1 × 105 mm. Vygon, Écouen, France). Collected blood samples were individually identified and stored in vacuum collection tubes (Vacutest^®^, Vacutest KIMA S.r.l., Arzergrande, Italy) at 5 °C for a maximum of 24 h before their processing. Once the blood separated into its components through coagulation process, the samples were centrifuged at 2000× *g* for 15 min, and the resulting blood sera were collected and stored at −20°C until analysis. In order to evaluate the samples’ quality, we measured the protein content of each sample with the use of an optical refractometer. The carcasses examined in 2018–2019 were also inspected to determine the tick infestation [47], and data on the hunting location were collected.

### 2.2. Serological Assays

The obtained sera were heat-inactivated at 56 °C for 30 min before testing. Serological analyses were performed with a commercial competitive immune enzymatic assay (EIA TBEV Ig, TestLine Clinical Diagnostics s.r.o., Brno, Czech Republic), according to the manufacturer’s instructions. This test is designed to measure the total antibody serum titer of all vertebrate species, except mice, against TBEV in serum; its diagnostic sensitivity and specificity were both declared as 95.7%. To confirm or exclude animal exposure to TBEV, borderline results were further subjected to a plaque reduction neutralisation test (PRNT) for TBEV, simultaneously testing for West Nile virus (WNV) and Usutu virus (USUV) to exclude the occurrence of cross-reactive antibodies. Roe deer sera originating from a recent survey on TBEV in the Netherlands [50], which were both positive in another commercial ELISA (Immunozym FSME IgG all species with inactivated TBEV coating; PROGEN Biotechnik GmbH), and in a TBEV serum neutralization test [51], were used as positive controls.

A day before performing the PRNT, 24-well cell culture plates were seeded with 2·105 A549 (human lung carcinoma) cells to obtain 80% confluence the next day. A twofold series dilution from 1:8 to 1:2048 was prepared with already inactivated borderline sera from wild ungulates. A virus solution containing 70 plaque-forming units was added in the same volume as the serum dilutions: TBEV ‘Salland’ [52], WNV (lineage 2, B-956 Uganda strain) and Usutu virus (virus isolate Netherlands 2016). After 1 h of incubation at 37 °C and 5% CO_2_, the virus–serum mixtures were added in duplicate to the 24-well plates. Serum, virus and cell controls were also added to each 24-well cell culture plate. After another hour of incubation at 37 °C and 5% CO_2_, an agar overlay (1:1, 2% SeaPlaque^TM^ Agarose (Lonza Bioscience, Geleen, the Netherlands) and MEM (Temin’s modification) (2X), no phenol red with 4% FBS, 2% penicillin/streptomycin and 5% HEPES, pH 7.4) was added. After four (TBE) or five (Usutu and West Nile) days the plates were fixed with 10% formaldehyde. The plates were then coloured with crystal violet (1% CV/20% ethanol) and washed two times with distilled water. When the plates were dry, plaques were counted. The PRNT50 value was defined as the highest serum dilution that reduced the number of plaques by 50% or more compared with the virus control.

### 2.3. Statistical Analyses

We determined a minimum sample size of 200 animals for testing by assuming a 1.5% minimum expected seroprevalence of TBEV antibodies in the wild ruminant population, with a 95% confidence level and 5% type I error. The expected prevalence was assumed based on data reported in literature, with seroprevalence ranging from 0.7% to 5.1% in non-endemic areas [53,54].

Prevalence of tick infestation, expressed as the number of animals presenting at least one tick, and 95% confidence intervals (CIs) were calculated for ungulate species and different altitudinal ranges. Mean, minimum and maximum protein concentrations were calculated for ungulates species and expressed in g/L.

## 3. Results

**Figure 1:**
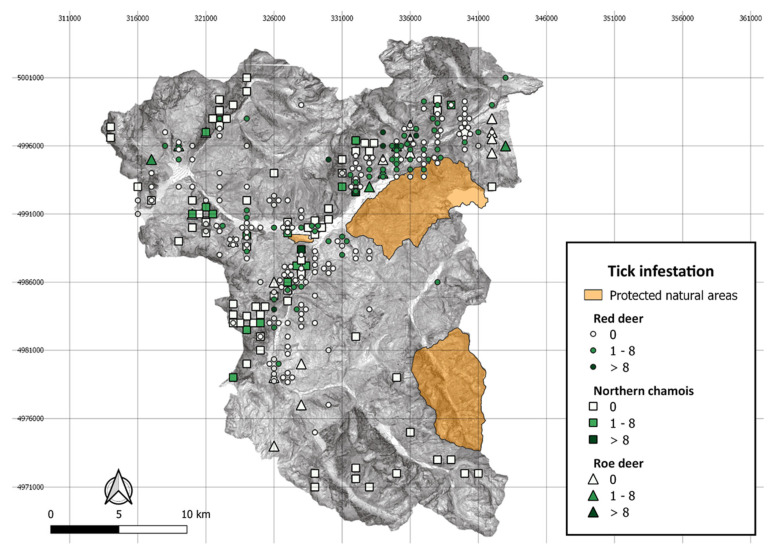
Distribution of culling locations within the hunting district by wild ruminant species and the number of *Ixodes ricinus* ticks collected per animal, high Susa Valley 2018–2019.

We collected a total of 268 sera from free-ranging wild ruminants, including 211 sera from animals inspected for ticks in 2018–2019 (Table 1) and 57 sera collected in 2017. In detail, 81 sera (30.2%) were collected in the 2018 hunting season, 130 (48.5%) in 2019, (Table 1) and 57 sera (21.3%) in 2017. These latter samples were collected from 5 red deer (*Cervus elaphus*) and 52 northern chamois (*Rupicapra rupicapra)*, and the serum samples collected in 2018–2019 were from red deer (n = 142), northern chamois (n = 64) and roe deer (*Capreolus capreolus*; n = 5). Among the red deer, 33.3% (95% CI = 25.6–41.8) of animals were infested by *I. ricinus* ticks and were shot at altitudes ranging between 750 and 2200 m; 20.3% (95% CI = 11.3–32.2) of chamois were parasitized and were culled between 1200 and 2200 m, and the two infested roe deer were shot at 1250 and 1600 m a.s.l. (Table 1; Figure 1). 

We failed to collect blood or obtained inadequate blood for testing from 126 hunted animals since hunted animals occasionally showed damage to the head region, hampering the blood extraction. Moreover, we excluded from testing sera with low quality (uncoloured sera with protein content values below 20 g/L). Data on wild ungulates subjected to serological testing is available in the Supplementary Material.

Through serological analyses by the EIA TBEV Ig test, 263 sera samples tested negative for TBEV, and the remaining five samples had borderline results. These latter samples were further confirmed to be negative for the presence of specific antibodies against TBEV, and also for WNV and USUV, by PRNT.

## 4. Discussion

Our survey indicates that TBEV was not circulating in the study area, at least in the investigated time interval, despite *I. ricinus* ticks being widespread and parasitising ungulates across a wide altitudinal range. While the absence of evidence is not evidence for absence, we hypothesize that, given the number of animals tested in relation to the size of the tick-suitable habitat in this area, the sampling effort was very thorough and higher than, for example in the Netherlands [4,50] and the UK [5], where they managed to identify several TBEV foci using fewer deer samples per surface area.

To assess the occurrence of TBEV, different surveillance approaches have been undertaken across Europe, each of them with advantages and shortcomings [48]. Molecular analyses in ticks provide direct evidence of TBEV occurrence but require greater research efforts due to the spotted distribution of the virus and its variable occurrence in different tick populations [55,56]. Indeed, large numbers of questing ticks are often required for TBEV detection, which in turn does not assure success in its detection, even in known endemic foci [57]. Surveillance based on reports of human cases may not reflect the real scenario either since most of the cases do not lead to clinical manifestations [58], thus resulting in the underestimation of cases. In addition, confirmed human cases usually indicate the place of residence and may not necessarily correspond to the area of infection, limiting the tracing of endemic TBEV risk areas [10].

Monitoring antibodies against the virus in domesticated and wild animals appears to be a more reliable strategy for detecting new TBEV foci, especially in areas where human cases have not yet been reported [37,54,59]. Recent studies underlined the role of large wildlife species in the epidemiology of TBEV [60]: they are maintenance hosts for tick populations, and although they are non-competent hosts for TBEV, they may contribute to the non-viremic transmission route between infected and uninfected ticks co-feeding on the same host [61,62]. Wild ruminants’ movements may also potentially introduce infected ticks into new areas [11,63], leading to the emergence of TBEV foci in naïve areas. In accordance with the patchy distribution of TBEV, seroprevalence studies in wild ungulates have disclosed varying results throughout Europe, ranging from 2% in roe deer from the Netherlands and Austria [4,59], up to around 42% of seroprevalence in moose from Norway [64]. Recently, very low seroprevalence levels in red deer (1.4%) and roe deer (0.7%) were detected in Norway [54]. Therefore, although our serological survey uncovered no evidence of TBEV circulation, we cannot rule out the presence of antibodies in the local wild ruminant population at a very low prevalence (below a maximum disease prevalence of 1.2%). Moreover, we have to consider the possibility of false negative results due to the test characteristics (up to 11 possible false negatives, based on test sensitivity) or sera quality. Nonetheless, the absence of positives is in accordance with the fact that, to date, there is no evidence of TBEV circulating in the area.

Hunted wildlife can be a cost and time-efficient source of samples for research and surveillance of pathogens [65]. However, blood sampling can be challenging in culled animals. We successfully collected blood from almost 70% of animals by endocranial venous sinuses puncture. Our success rate is lower than that reported by other authors [49,66] who employed the same sampling method in wild ruminants and wild boar. This may be explained by differences in the culling-to-sampling time interval and carcass conditions. In particular, Jiménez-Ruiz et al. [49] sampled carcasses that had not been eviscerated at the sampling time, which probably ensured a smaller quantity of blood loss. By contrast, our carcasses occasionally showed damages in the head region, caused by bullet impact and/or fractures due to falling from cliffs, thus probably resulting in blood loss and consequently hindering its sampling. Moreover, for hygienic reasons, hunters are used to delivering carcasses already blood-drained and eviscerated to the check station. Finally, the elapsed time between field dressing and carcass delivery is quite irregular and often spans several hours.

Regarding the quality of our sera, their mean protein content values were similar to those previously reported in different wild ruminant species [67,68,69,70,71], even though our use of an optical refractometer is not the most accurate measuring technique. When discussing the variability of protein content, however, we should also consider the sampling/capture method used, the season of sampling and the health status of the animals. For instance, Poljicak-Milas et al. [68] collected blood samples during another season (January to March) using different sampling methods according to whether the deer were alive or not. Rosef et al. [69], on the other hand, collected blood from deer that were chemically immobilized, which may alter the biochemical components in the serum [72]. The mean protein values of our northern chamois sera were comparable to values reported in Spain for southern chamois (*R. pyrenaica*), although the latter were collected in vivo during the spring–summer period [70].

The needle length used for blood extraction may also affect the quality of the blood serum. In fact, we observed some anomalies in around one-third of the sera collected, whose protein content was below 20 g/L. These samples yielded uncoloured sera after blood clotting, which may be attributable to some degree of unintentional blood dilution with cerebrospinal fluid taken from the cranial cavity. Similar shortcomings were also observed by Jiménez-Ruiz et al. [49], who reported unsuccessful blood extractions using an 80-mm-long needle in European mouflon (*Ovis aries musimon*). Notwithstanding, endocranial venous sinuses puncture allowed blood collection in most of the animals investigated, despite the challenging field conditions.

## 5. Conclusions

Our serosurvey indicates the absence of TBEV in the Susa valley, northwestern Italian Alps. However, our study was limited to a small area of the western Alps, and to the period 2017–2019. The need for large-scale routine surveillance in Piedmont region and other naïve areas of Italy is supported by the increase in TBE cases in Europe and by the identification of new endemic areas. In this respect, serosurveillance in wild and domestic ruminants exposed to ticks could be a useful tool for the early detection of TBEV foray. In our study area, where large mammals are routinely subjected to population control plans (hunted game) or sampled for other purposes (sanitary controls in livestock in Alpine pastures), such sampling would be particularly convenient in terms of time and cost savings.

## Figures and Tables

**Table 1:** The number of sera examined for TBEV for each wild ruminant species investigated, indicating the average content of serum proteins, the altitudinal range of the culling locations and the percentage of tick-infested individuals; high Susa Valley, Piedmont, 2018–2019.

Ungulate Species	Altitudinal Range (m a.s.l.)	Tick Infestation (%) [95% CI]	N Sera Tested	Mean Protein Concentration (g/L) [Min.–Max.]
Red deer ^1^ (*Cervus elaphus*)	750–1200	32.6 [19.1–48.5]	43	52.3 [20–115]
	1201–1600	37.9 [26.2–50.7]	66	
	1601–2000	23.1 [9.0–43.6]	26	
	>2000	33.3 [4.3–77.7]	6	
Northern chamois ^1^ (*Rupicapra rupicapra*)	800–1200	33.3 [4.3–77.7]	6	46.2 [20–100]
	1201–1600	37.5 [18.8–59.4]	24	
	1601–2000	6.7 [0.2–32.0]	15	
	>2000	5.3 [0.1–26.0]	19	
Roe deer (*Capreolus capreolus*)	1200–1600	40.0 [5.3–85.3]	5	60.3 [30–91]

^1^ Tested sera from red deer and chamois (n = 57) collected during 2017 are not included in the table because we lacked information about culling location and tick infestation. The sum of red deer sera in the table is 141 (instead of 142) because the hunting location of one animal culled in 2018 was missing.

## Data Availability

The data presented in this study are available in the Supplementary Material.

## Supplementary Materials

The following supporting information can be downloaded at: https://www.mdpi.com/article/10.3390/life12111888/s1, Table S1. Wild ungulates subjected to serological testing against Tick-borne encephalitis virus, High Susa Valley, northwestern Italy, hunting seasons from 2017 to 2019.
